# The impact of hunger on indulgent food choices is moderated by healthy eating concerns

**DOI:** 10.3389/fnut.2024.1377120

**Published:** 2024-08-23

**Authors:** Tobias Otterbring, Michał Folwarczny, Agata Gasiorowska

**Affiliations:** ^1^Department of Management, University of Agder, Kristiansand, Norway; ^2^Discipline of Marketing, J.E. Cairnes School of Business and Economics, University of Galway, Galway, Ireland; ^3^Faculty of Psychology in Wroclaw, SWPS University, Wrocław, Poland

**Keywords:** hunger, consumer choice, food choice, indulgence, healthy eating concerns

## Abstract

Research has shown that hungry individuals are more impulsive, impatient, and prone to make indulgent food choices compared to their satiated counterparts. However, the literature is still mixed, with some studies showing such results while others fail to demonstrate hunger effects on consumers’ choice behavior. The current cross-sectional study (*N* = 461) sought to address these inconsistencies by examining whether the link between hunger and people’s propensity to make indulgent (vs. virtuous) food choices is moderated by their healthy eating concerns. Our findings revealed a weak but significant association between participants’ self-reported hunger levels and their likelihood of making indulgent rather than virtuous food choices (e.g., preferring a chocolate cake instead of a fruit salad). Importantly, this effect was moderated by their healthy eating concerns, such that the link between hunger and choice likelihood of indulgent food options only emerged among participants who scored lower, but not higher, in healthy eating concerns. We also replicated these results in a robustness check that focused on the extent to which participants indicated having a healthy lifestyle (e.g., exercising regularly), with a similar moderating influence of this factor. Together, these findings shed light on the importance of considering certain boundary conditions for establishing a link between hunger and consumers’ food choices, thus adding nuance to the growing body of hunger-related literature. The results emphasize the importance of ensuring the availability of healthier snack options in environments wherein foods and beverages can be consumed, particularly at times when consumers tend to be hungry, to promote healthier eating habits.

## Introduction

Becoming hungry after skipping certain meals (e.g., breakfast) can have several costly consequences. For instance, hunger has been linked to individuals becoming “hangry” and easily irritated (1, 2) as well as greedy and seemingly selfish (3–5). Further, prior research attests that hungry individuals buy more food when shopping for groceries (6) and exhibit increased acquisition-related tendencies in terms of their desire to acquire both food and non-food objects (7), while simultaneously rating food in general, and calorie-dense dishes in particular, more favorably (8).

However, the literature is mixed with respect to the impact that hunger exerts on consumers’ decisions and choice behavior, with some studies showing hunger effects (9–13) and others demonstrating either negligible effects or no noticeable effects at all (14–20). One reason for these mixed results might be certain “hidden moderators” (21–23), with a given previously omitted variable potentially explaining why a certain relationship does not always hold. Indeed, hunger effects are often contingent on a wide variety of other factors, such as individual differences in self-control (24), impulsiveness (25), emotional eating (26), and consumers’ weight status (27).

Hunger is a physiological need for food, driven by the body’s requirement for energy and nutrients, often described as accompanied by stomach growls, feelings of emptiness, and a loss of energy (28). In contrast, cravings are intense desires for specific foods (e.g., the desire to consume something sweet), often influenced by psychological factors or sensory stimuli, and can occur independently of hunger (29). Thus, hunger refers to bodily needs, whereas cravings refer to wants and desires that can occur even without experiencing hunger.

Drawing on research on self-control conflicts (30, 31) and the role of visceral states, such as hunger, thirst, and sexual desire, in shaping intertemporal choice outcomes (32, 33), we theorize that the link between hunger and indulgent food choices [i.e., the selection of food options that are primarily consumed for their immediate pleasure, but that are typically unhealthy; Hildebrand et al. (34)] is contingent on aspects associated with healthy eating concerns. The link between hunger and a preference for indulgent foods is fairly well-established (35–46) and people often infer that virtuous foods [i.e., foods perceived to be healthy rather than tasty; (47)] will not satiate them to the same extent as indulgent foods (48). This conflict occurs because people often hold multiple goals when choosing what to eat: wanting to maintain their health and feeling full, thus posing a self-control conflict (49, 50). In fact, the association between eating healthy and feeling hungry can be so strong that it is directly associated in memory (51).

This effect is particularly pronounced among those who are not concerned with watching their weight (52). Indeed, there are individual differences in how concerned people are with eating healthily and watching their weight, as evidenced by a large body of research on restrained eating [e.g., Herman and Polivy (50) and Ward and Mann (53)]. Thus, it is possible that concern with healthy eating and maintaining a healthy lifestyle will moderate the impact of hunger on food choices such that those who do not tend to prioritize healthy eating or having a healthy lifestyle will pick more indulgent foods, whereas people who prioritize healthy eating and a healthy lifestyle show no difference. This pattern may occur because people who are used to consuming healthy foods or prioritizing a healthy lifestyle likely understand that eating healthy food is also satisfying and satiating; therefore, they are less likely to infer that indulgent foods are the only foods that satiate them (52, 54–56). Accordingly, we hypothesize that:

***H1:*** More (vs. less) hungry individuals are increasingly inclined to make indulgent (vs. virtuous) food choices.

***H2:*** Healthy eating and healthy lifestyle concerns moderate the link between hunger and indulgent (vs. virtuous) food choices, and only affects people who score lower but not higher in healthy eating and healthy lifestyle concerns.

## Method

The study included 461 undergraduates (49% female; *M*_age_ = 22 years) from a Northern European university, after excluding two additional participants who did not reply to the hunger items. No further demographic variables or individual characteristics beyond gender and age were collected, such as ethnicity, racial background, body mass index (BMI), or weight status, and the project received ethical approval by the Ethics Committee for Research at one of the authors’ universities (No. 03/E/06/2024). Our sample size has a statistical power greater than 99% to detect moderate effect sizes equivalent to *r* = 0.20 or *d* = 0.40, assuming two-tailed tests and the conventional alpha level of *α* = 0.05. In fact, our sample size gives us close to 80% statistical power to detect an attenuated interaction, wherein the effect size for the link between hunger and indulgent food choices corresponds to *d* = 0.50 among participants with lower healthy eating concerns, and with no such link for participants with higher healthy eating concerns, assuming two-tailed tests and the same alpha level (57). Considering that effect sizes of *r* = 0.20–0.25 or *d* = 0.40–0.50 are the typical ones in the published psychology and consumer behavior literature (58–61), our study is appropriately powered for testing our focal hypotheses.

Data collection took place at different times during a series of consecutive days, with participants recruited in the vicinity of a university cafeteria. The decision to collect data at different times was made to get more heterogeneity in participants’ self-reported hunger levels. As such, participants did not receive any instructions regarding food consumption or food restriction prior to taking part in the study, and the data were collected both in connection to breakfasts, lunches, and afternoon snacking breaks as well as between such meals.

Participants were verbally presented with a set of eight binary food choices from Otterbring (62) and were asked to choose the alternatives that appealed to them the most on without accompanying visualizations or photographs of the different food options and with no direct reference to liking or eating; see Appendix A1 for the items used to capture each of our focal constructs. Each food choice included one virtuous option and one indulgent alternative, such as between fruit salad (A) and chocolate cake (B). Items were rated on a 7-point scale (1 = definitely A; 7 = definitely B) and an index variable was calculated by averaging the responses (*α* = 0.76), with higher values representing more indulgent food choices. Participants also replied to six statements intended to measure hunger on a 7-point scale (1 = strongly disagree; 7 = strongly agree) from Otterbring (62), with such scale formats commonly used to measure hunger across disciplines [e.g., Epstein et al. (63), Petit and Otterbring (64), Suher and Raghunathan (65), and Wang and Park (66)]; see Appendix A1 for items. However, a factor analysis using direct oblimin revealed that these items loaded on two separate factors with eigenvalues above 1, with a dominant first factor explaining most of the variance (>50%). This first factor, which contained four of the six times, served as our predictor, with its items averaged into a composite hunger index (*α* = 0.88). The remaining two items that belonged to the second factor only dealt with a desire to consume something sweet, which might reflect cravings rather than hunger *per se*. This construct validity concern, combined with the fact that the second factor did not correlate with the dominant first factor more than moderately (*r* = 0.33, *p* < 0.001) and only added incrementally to the explained variance, led us to focus on the dominant first factor to facilitate parsimonious analyses.

In the end of the survey, participants replied to items measuring healthy eating concerns and, as a robustness check, two variables meant to capture a healthy lifestyle. To measure healthy eating concerns, participants stated their agreement on each of the six items (e.g., It is important to me that the food I eat on a typical day: keeps me healthy; is nutritious) comprising the health factor in the food choice questionnaire (67); see Appendix A1 for all items. Items were rated using the same 7-point response format as for the hunger items and an index of healthy eating concerns was calculated by averaging the responses on each of these items (*α* = 0.85). Similarly, a healthy lifestyle index was computed by averaging participants’ responses on the two items: “I exercise regularly” and “I have a healthy lifestyle,” still using the same response format (*r* = 0.68, *p* < 0.001).

### Validation study

A smaller validation study on 37 undergraduates (43% female; *M*_age_ = 24 years) was conducted to ensure that the items in each food choice differed on a set of dimensions associated with indulgence. For each binary choice, participants were asked to indicate the most affective, immediate, vice, and impulsive option, and hence leave the most cognitive, delayed, virtuous, and deliberate option. A Pearson’s chi-square analysis revealed that participants correctly classified the indulgent food items to a significantly higher extent than what can be assumed by chance [*M* = 81%, *χ*^2^ (1) = 21.26, *p* < 0.001]. Thus, the food items were deemed appropriate for use in the main study.

## Results

### Main analysis

**Figure 1:**
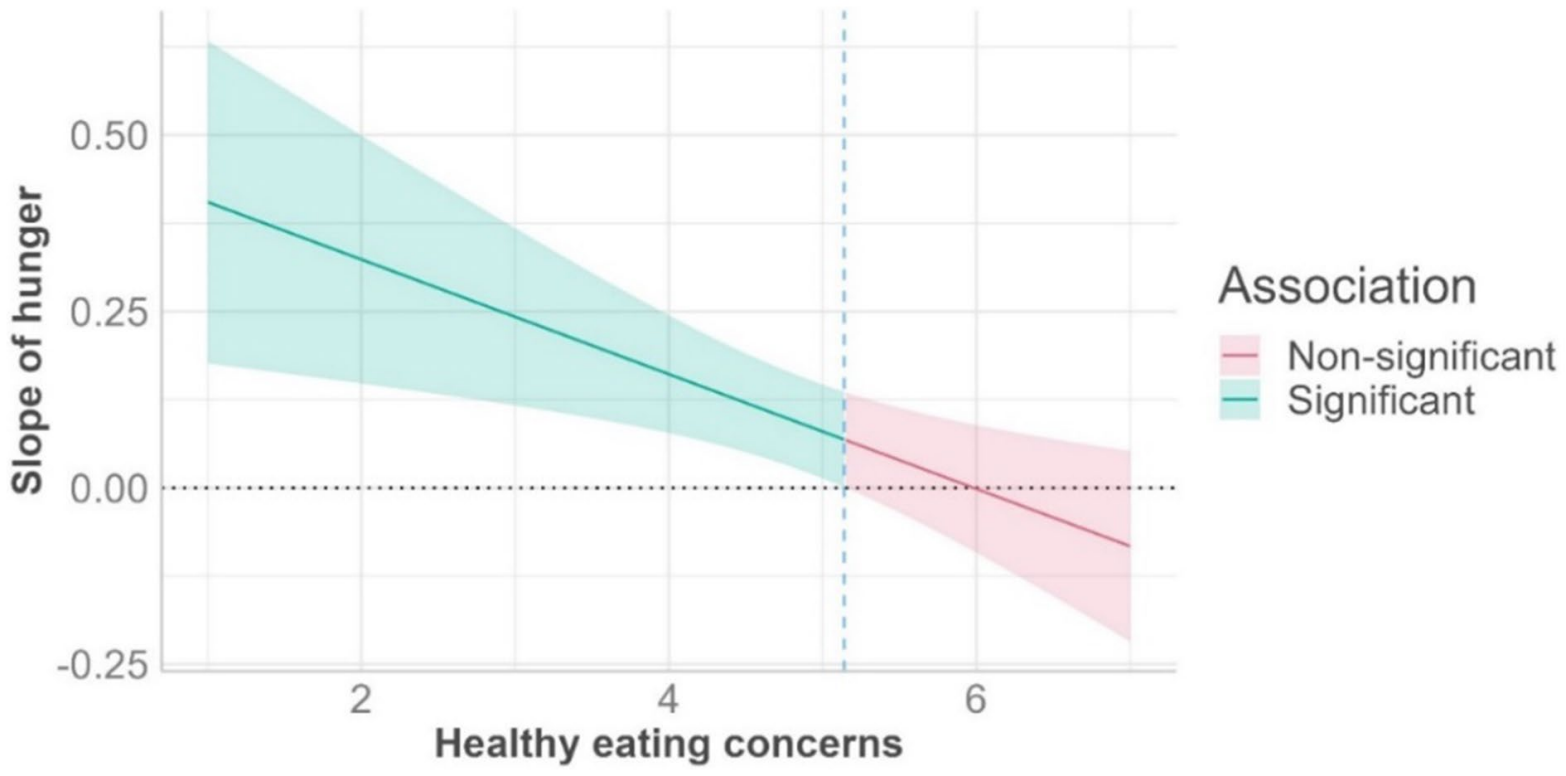
Indulgent food choices as a function of hunger and healthy eating concerns. The slope of hunger (*y*-axis) significantly predicts indulgent food choices at lower, not higher, levels of healthy eating concerns.

To examine whether hunger was linked to more indulgent food choices (**H1**) and whether healthy eating concerns moderated this presumed pattern (**H2**), we conducted a simple moderation analysis [PROCESS Model 1; Hayes (68)]. Hunger (continuous) was the predictor, healthy eating concerns (continuous) acted as the moderator, and indulgent food choices (continuous) constituted the outcome variable. As regression-based analyses, including those conducted in PROCESS, are robust to nonnormal errors in estimation (69), we retained our original analytic approach, although most of our included variables were not normally distributed.^1^ The overall model was statistically significant and explained roughly 11% of the variance in participants’ indulgent food choices, *F* (3, 457) = 18.12, *p* < 0.001, *R*^2^ = 0.106. Consistent with **H1**, the link between hunger and indulgent food choices was significant and positive (*b* = 0.10, *t* = 2.84, *p* = 0.005) suggesting that hungry (vs. satiated) participants are more inclined to make indulgent food choices. The link between healthy eating concerns and indulgent food choices was significant and negative (*b* = −0.36, *t* = −6.76, *p* < 0.001); specifically, participants who score higher (vs. lower) on the healthy eating concerns index are less likely to choose indulgent food options. However, in line with **H2**, the link between hunger and indulgent food choices was moderated by participants’ healthy eating concerns (*b* = −0.08, *t* = −2.85, *p* = 0.005). As the moderator was a continuous variable, we performed a “floodlight analysis” to better understand the nature of the moderation (70). The moderator value at which the interaction becomes significant, known as the Johnson-Neyman point, was a mean-centered value of 0.35 (*Z* = 1.96; *p* = 0.050). This means that the hunger effect on indulgent food choices was significant for 59.65% of participants whose mean-centered value on the health factor was equal to or below 0.35 (corresponding to a mean value of 5.15). Thus, hunger was positively associated with the likelihood of making indulgent food choices, but only among participants with lower (not higher) levels of healthy eating concerns; see Figure 1.

### Robustness check

**Figure 2:**
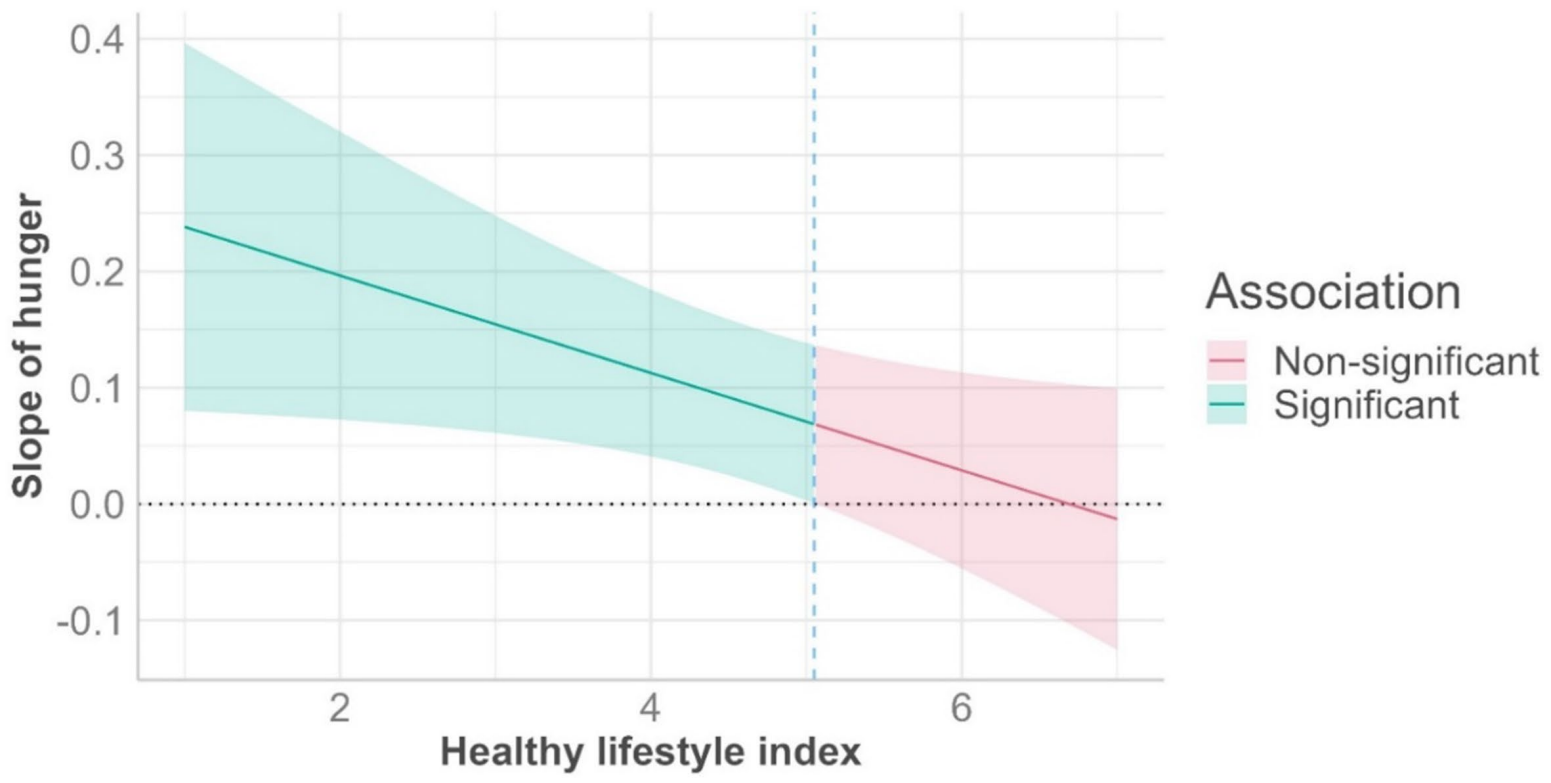
Indulgent food choices as a function of hunger and a healthy lifestyle. The slope of hunger (*y*-axis) significantly predicts indulgent food choices at lower, not higher, levels of the healthy lifestyle index.

As a robustness check, we performed a similar moderation analysis, with the health factor replaced by our healthy lifestyle index as the moderator. This analysis produced comparable results. The overall model was statistically significant and explained roughly 9% of the variance in participants’ indulgent food choices, *F* (3, 457) = 15.30, *p* < 0.001, *R*^2^ = 0.091. First, in further support of **H1**, the link between hunger and indulgent food choices was significant and positive (*b* = 0.08, *t* = 2.50, *p* = 0.013). Second, the link between the healthy lifestyle index and indulgent food choices was significant and negative (*b* = −0.23, *t* = −6.14, *p* < 0.001); participants scoring higher (vs. lower) on this variable were less likely to choose indulgent food options. However, consistent with our main analysis and **H2**, the link between hunger and indulgent food choices was moderated by the healthy lifestyle index (*b* = −0.04, *t* = −2.11, *p* = 0.035). As the moderator was a continuous variable, we performed another “floodlight analysis.” The moderator value at which the interaction becomes significant was a mean-centered value of 0.40 (*Z* = 1.96; *p* = 0.050). This means that the hunger effect on indulgent food choices was significant for 58.79% of participants whose mean-centered value on the healthy lifestyle index was equal to or below 0.40 (corresponding to a mean value of 5.06). Thus, hunger was positively associated with the likelihood of making indulgent food choices, but only among participants with a less (not more) healthy lifestyle; see Figure 2 and Table 1 for the zero-order correlations between our studied constructs.

**Table 1:** Grand means (*M*), standard deviations (*SD*), and zero-order correlations between studied variables.

Construct	*M* (*SD*)	Indulgent food choices	Healthy eating concerns	Healthy lifestyle
Hunger	3.70 (1.84)	0.10*	0.06	0.05
Indulgent food choices	4.21 (1.40)	1	−0.28***	−0.27***
Healthy eating concerns	4.80 (1.17)	–	1	0.50***
Healthy lifestyle	4.66 (1.67)	–	–	1

*p < 0.05; **p < 0.01; ***p < 0.001.

## Discussion

This research investigated the effects of hunger on preferences for indulgent relative to virtuous food options. The findings revealed a positive link between hunger and the preference for indulgent foods; the hungrier individuals were, the more they preferred indulgent foods. However, this association was moderated by healthy eating concerns and healthy lifestyle habits. Specifically, among participants who exhibited lower concern for healthy eating and indicators of a less healthy lifestyle, this association was significant and positive. By contrast, hunger did not predict the likelihood of making indulgent food choices among participants who were increasingly concerned with healthy eating and maintained a healthier lifestyle.

Together, the current work builds on and contribute to the existing body of research concerning the dynamic interplay between hunger and preferences for indulgent food, with a novel exploration into the moderating roles of healthy eating concerns and lifestyles. This addition to the literature offers a novel lens through which hunger influences food preferences, particularly against the backdrop of mixed findings from past research. Our investigation into these moderators not only extends prior related research but also illuminates the conditions under which hunger produces an increased inclination to choose and prefer indulgent foods. This is especially relevant given that numerous studies in the domain of food-related research have reported mixed effects, often attributed to the lack of consideration for pivotal moderating factors [e.g., Folwarczny et al. (71) and Aksakalli Bayraktar et al. (72)]. By weaving in the perspectives of healthy eating concerns and lifestyles as critical to shaping the hunger-indulgence link, this research advances previous theoretical models and offers a more nuanced understanding of when hunger is linked to indulgence in the food domain. In so doing, we offer valuable insights for theoretical developments and practical applications in dietary behavior and health interventions.

Considering numerous calls for improving diet quality globally [e.g., Willett (73)], the current findings offer several recommendations to mitigate the negative impact of hunger on the nutritional quality of the foods people consume. First, as the link between hunger and indulgent food choices was only significant among participants who were relatively unconcerned with their eating and lifestyle activities associated with health, promoting healthy habits is paramount. This is particularly true given that hunger—as an inevitable and evolutionarily informed factor—influences food preferences (74, 75). Second, considering the limited effectiveness of promoting eating certain habits and lifestyle changes in modifying consumer attitudes and behavior, it may be more beneficial to encourage diets in which meal timing is aligned with the human circadian rhythm, thereby minimizing experiences of hunger (76). Third, consumers may be particularly prone to temptation when they are hungry. Thus, ensuring that virtuous options are accessible, financially and otherwise, at the point of purchase could be advantageous. Indeed, such strategies have proven successful in promoting healthier food choices (77).

### Limitations and future research

Our study has some limitations that should guide future research directions in this domain. First, instead of manipulating hunger levels, we employed a cross-sectional research design, which complicates causal inferences (78). Relatedly, we did not ask participants for the reasons behind their current state of hunger or satiety. It is plausible that intentional hunger due to dietary restrictions, such as following an intermittent fasting diet, results in different food preferences than those shaped by unintentional hunger (e.g., a lack of time). Research has linked unintentional hunger to preferences for foods often associated with weight gain (79, 80), which may not be the case when someone intentionally skips breakfast. Thus, future research should examine the reasons behind experiencing hunger and utilize experimental paradigms to establish causal relationships between our studied constructs.

Second, our study utilized a student sample, which may be viewed as a form of convenience sampling, highlighting the need for sample diversification (81, 82). Indeed, university students may generally be healthier than the average population due to their age, fitness levels, and knowledge of health and nutrition, thus calling for future research using more diverse samples to test the robustness and replicability of our findings across populations and contexts (83, 84). Moreover, we did not collect basic demographics beyond gender and age, meaning that other unmeasured demographic variables could have played some role in shaping our findings (e.g., ethnicity, racial background, BMI, or weight status).

Third, the ecological validity of our results is debatable, as our focal dependent variable was restricted to hypothetical choices beteween indulgent and virtuous food options, potentially leading to spurious effects (85–87). Recent studies in food-related fields also indicate that certain measures may be effective in artificial contexts (e.g., inside the walls of a lab) but less so in more realistic settings, such as measuring willingness to pay in a local currency (88).

Fourth, individual factors such as high emotional eating can influence hunger responses. For example, high emotional eaters demonstrate higher levels of hunger when confronted with stressful situations, with this pattern being more pronounced if they are unable to control their impulses [i.e., low control; van Strien et al. (26)]. Customers also differ in terms of the value they place on internal and external cues that shape their food preferences (89). Therefore, future research should seek potential alternative moderators of the effects reported herein.

Fifth, we did not measure actual food intake or habitual eating behavior, which could provide more comprehensive insights into dietary patterns. Self-reported preferences or stated food intake can differ substantially from actual intake. Past research has found that people tend to underreport their energy intake by as much as 20% (90). Therefore, additional research should measure participants’ actual food intake under conditions of hunger.

Finally, the dimension of virtuous versus indulgent foods is relatively new in food science (91, 92), necessitating further exploration to establish a comprehensive network of relationships between this dimension and other variables.

## Conclusion

Conventional wisdom suggests that hunger leads to indulgent (pleasure-focused) rather than virtuous (healthy) food choices. However, the academic literature into the effects of hunger on specific food choices is mixed. This study found that hungry participants were more likely to choose indulgent foods compared to their satiated counterparts. This effect was moderated by healthy eating concerns: participants with lower concerns showed this tendency, whereas those with higher concerns did not. These findings highlight the importance of educating the public about healthy eating, as hunger is sometimes unavoidable due to factors such as a lack of time for breakfast.

## Data availability statement

The raw data supporting the conclusions of this article will be made available by the authors, without undue reservation.

## Ethics statement

This project received ethical approval by the Ethics Committee for Research at the SWPS University (No. 03/E/06/2024). All participants voluntarily agreed to take part in the anonymous survey. Written informed consent was not required in accordance with the national legislation and institutional requirements for non-medical research.

## Author contributions

TO: Conceptualization, Formal analysis, Methodology, Project administration, Writing – original draft, Writing – review & editing. MF: Conceptualization, Visualization, Writing – review & editing. AG: Conceptualization, Validation, Writing - review & editing.

## Footnotes

^1^As evidenced by Kolmogorov–Smirnov tests (hunger: *p*<0.001; healthy eating concerns: *p*=0.055; healthy lifestyle: *p*<0.001; indulgent food choices: *p*<0.001).

## Appendix A1 Items used to capture the key constructs

### Hunger (1 = Strongly disagree; 7 = Strongly agree)

1. I need to do something about my hunger.
2. I would like to have something appetizing right now.
3. Right now, I feel very hungry.
4. It feels like I have good appetite right now.
5. I would need something to satisfy my need for sweetness.*
6. I’m craving something sweet.*

Note: * = Items discarded after factor analysis.

### Virtuous vs. Indulgent food choices (1 = Definitely A; 7 = Definitely B)

1. A = Apple vs. B = Candy
2. A = Carrots vs. B = Chocolate
3. A = Sparkling Water vs. B = Soda
4. A = Chicken Salad vs. B = Hamburger with French Fries
5. A = Fruit Salad vs. B = Chocolate Cake
6. A = Sliced Vegetables vs. B = Potato Chips
7. A = Unsalted Walnuts vs. B = Cheese Doodles
8. A = Orange vs. B = Pick & Mix (Candy)

### Healthy eating concerns (1 = Strongly disagree; 7 = Strongly agree)

It is important to me that the food I eat on a typical day:

1. Contains a lot of vitamins and minerals.
2. Keeps me healthy.
3. Is nutritious.
4. Is high in protein.
5. Is good for my skin/teeth/hair/nails etc.
6. Is high in fiber and roughage.

### Healthy lifestyle (1 = Strongly disagree; 7 = Strongly agree)

1. I exercise regularly.
2. I have a healthy lifestyle.
